# Psycholinguistic norms for the dominant and secondary names of 700 LinguaPix color photographs in Mandarin Chinese

**DOI:** 10.3758/s13428-025-02644-z

**Published:** 2025-04-11

**Authors:** Ya-Ning Chang, Leqi Cheng, Jie Wang, Yiu-Kei Tsang, Agnieszka Ewa Krautz, Susanna Siu-sze Yeung, Suiping Wang, Hsuan-Chih Chen

**Affiliations:** 1 Miin Wu School of Computing, National Cheng Kung University, Tainan, Taiwan; 2 Department of Psychology, The Education University of Hong Kong, Hong Kong S.A.R, 10 Lo Ping Road, Taipo, N.T China; 3 Department of Education Studies, Hong Kong Baptist University, Hong Kong, S.A.R. China; 4 Department of English, American and Celtic Studies, University of Bonn, Bonn, Germany; 5 Philosophy and Social Science Laboratory of Reading and Development in Children and Adolescents (South China Normal University), Ministry of Education, Guangzhou, China; 6 Department of Psychology, The Chinese University of Hong Kong, Hong Kong, S.A.R. China

**Keywords:** Picture naming, Norms, Chinese, Mandarin, Secondary name

## Abstract

Norming studies on picture naming usually identify different correct names for each picture and provide more information on the dominant name (i.e., the most frequently produced name of a given picture) or weighted values based on all the names. The current study is among the first attempts to establish psycholinguistic norms for both the dominant and secondary names of pictures. The following norms in Mandarin Chinese were provided for 700 color photographs from the LinguaPix database: name agreement, naming latency, name length, image agreement, age of acquisition (AoA), and concept familiarity of the dominant and secondary names, as well as overall accuracy, number of names, *H*-statistic, familiarity, visual complexity, valence, and arousal of the pictures. This dataset increases the diversity of stimuli available for picture naming studies in Chinese and greatly facilitates stimuli selection by allowing researchers to manipulate not only common psycholinguistic properties of the dominant picture name but also those of the secondary name and the relations between them. The database is available in the Open Science Framework repository (https://osf.io/5rphx/).

## Introduction

Picture naming is a widely employed paradigm in research that enables the investigation of cognitive processes involved in lexical retrieval and language production. Commonly, it requires participants to generate a spoken or written/typed name for a given picture. In turn, measures of accuracy and response time, derived from the naming process, are thought to illuminate the underlying processing of pictures and their names.

## Factors influencing picture naming performance

Various characteristics of pictures have been demonstrated to influence naming speed and accuracy. For instance, studies have consistently shown that pictures with high *name agreement* elicit more accurate and rapid responses (Alario et al., 2004; Barry et al., 1997; Bonin et al., 2002; Cuetos et al., 1999; Ellis & Morrison, 1998; Liu et al., 2011; Snodgrass & Yuditsky, 1996; Zhong et al., 2024). This suggests that when most people agree on the name of the picture, the concept of the picture becomes more readily distinguishable and accessible. *Image familiarity* is also an important variable, wherein individuals tend to generate accurate and quick responses towards pictures that are familiar to them (Cuetos et al., 1999; Johnston et al., 2010; Krautz & Keuleers, 2022). In terms of *visual complexity*, i.e., the degree of detail depicted by the image, some studies have demonstrated that a visually complex picture tends to require longer response time (Alario et al., 2004; Ellis & Morrison, 1998) while others have failed to find such an effect (Bonin et al., 2002, 2003; Snodgrass & Yuditsky, 1996). This discrepancy could be due to the fact that the use of simple black-and-white drawings does not always pose strong visual difficulties (Alario et al., 2004). Other factors, such as emotional states triggered by pictures (*valence* and *arousal*), have also been demonstrated to affect picture naming performance, although the findings have been inconclusive (Blackett et al., 2017; Krautz & Keuleers, 2022). Further investigations are necessary to gain a comprehensive understanding of the potential impact of affective factors on lexical retrieval.

Moreover, the impact of psycholinguistic factors related to the names of pictures rather than the image have also been investigated. Studies have demonstrated a robust effect of *age of acquisition* (*AoA*) on the naming performance, in which names of pictures that were learned early are processed more quickly and accurately than those learned later (Alario et al., 2004; Barry et al., 1997; Ellis & Morrison, 1998; Liu et al., 2011; Snodgrass & Yuditsky, 1996; Zhong et al., 2024). It is likely that semantic representations are more robust for picture names learned earlier. As a picture could have multiple concept names, another crucial psycholinguistic factor in picture naming is *image agreement*, which measures how closely a given picture resembles an individual’s mental image of one concept name. Naming latencies for pictures with high image agreement are shorter than those with low image agreement (Alario et al., 2004; Barry et al., 1997; Liu et al., 2011; Zhong et al., 2024). Finally, *concept familiarity*, measuring the degree of familiarity with a concept name of a given picture, also impacts picture naming performance (Liu et al., 2011; Zhong et al., 2024). Individuals respond more quickly when producing familiar concept names of pictures relative to unfamiliar names.

### Norming studies on picture naming

In the picture naming literature, numerous normed picture datasets have been constructed. The widely used standardized set of black-and-white line drawings was provided by Snodgrass and Vanderwart (1980), wherein four key variables were standardized in English, including naming agreement, image agreement, familiarity, and visual complexity. The norming process has been largely adopted in creating normed pictures for European language systems (Barry et al., 1997; Bonin et al., 2002; Cuetos et al., 1999; Pind et al., 2000) as well as Asian language systems (Weekes et al., 2007). While studies using line drawings have revealed important cognitive processes underlying picture naming, these stimuli lack variations in physical properties such as color, texture, and luminance that are potentially important for triggering sensorimotor and affective aspects of conceptual processing (e.g., van Hoef et al., 2024). Therefore, color pictures have been introduced. They tend to result in better object recognition and higher name agreement than line drawings (Rossion & Pourtois, 2004). A color version of Snodgrass and Vanderwart (1980) pictures has also been created and normalized (Bakhtiar et al., 2013; Bonin et al., 2013; Dimitropoulou et al., 2009; Raman et al., 2014; Rossion & Pourtois, 2004; Weekes et al., 2007). Furthermore, larger numbers of line drawings (Alario & Ferrand, 1999; Bonin et al., 2003; Cycowicz et al., 1997; Liu et al., 2011; Nishimoto et al., 2005) and color line drawings or pictures (Brodeur et al., 2010, 2012; Moreno-Martínez & Montoro, 2012; van Hoef et al., 2024; Zhou & Chen, 2017) have been developed in order to support experimental manipulations and application in a wider range.

Cross-language picture norms have been developed to control for potential variations in picture manipulations across studies (Bates et al., 2003; Brodeur et al., 2012; Duñabeitia et al., 2018; Krautz & Keuleers, 2022; Szekely et al., 2004). For example, Duñabeitia et al. (2018) developed the MultiPic norm dataset for six European languages, comprising 750 colored drawings of objects. In their subsequent study (Duñabeitia et al., 2022), a subset of 500 colored drawings was extended to 32 different languages. Another more recent multi-language picture norm is the LinguaPix database (Krautz & Keuleers, 2022), originally providing 1547 color photographs spanning 42 semantic categories in German, and subsequently extended to English, Polish, and Cantonese Chinese in their online database (https://linguapix.uni-mannheim.de/frontend/web/index.php; Krautz, 2023).

While the development of most multi-language picture norms generally starts from European languages, there has been growing interest in extending the norms to more distant languages. Such a norm could enable researchers to investigate commonalities and differences in picture naming across a wide range of language systems. For example, the latest version of MultiPic norm has included Mandarin Chinese, Japanese, and Korean (Duñabeitia et al., 2022; see a Cantonese Chinese version in Zhong et al., 2024). With these norms, Duñabeitia et al. (2022) demonstrated several important variations across languages (e.g., the modal response percentage ranging from 73.3% for Mandarin Chinese to 93% for Spanish).

### The current study

The current study is among the attempts to establish psycholinguistic norms for a set of pictures in one of the most widely spoken languages, Mandarin Chinese. So far, existing databases have provided Mandarin norms for black-and-white line drawings (Bates et al., 2003; Liu et al., 2011) and colored line drawings (Duñabeitia et al., 2022). The current study aimed to extend this line of research by creating a Mandarin dataset for color photographs (i.e., a subset of LinguaPix pictures; Krautz & Keuleers, 2022), which would increase the diversity of stimuli available for picture naming studies in Chinese.

The LinguaPix database (Krautz & Keuleers, 2022) included a large set of color photographs depicting concrete and imageable objects across various semantic categories (e.g., food, animals, body parts, vehicles, professions, furniture). The photographs were edited to remove the initial background as well as any visible text or logos, making them optimal for experimental research in psycholinguistics and cognitive psychology. Although a set of Cantonese Chinese norms had been included in the LinguaPix database (Krautz, 2023), the current study still constituted a notable addition to the literature. Being the official language of Mainland China and Taiwan, Mandarin is the most widely spoken Chinese dialect (about 955 million native speakers; retrieved from https://www.worlddata.info/languages/chinese.php on February 9, 2025). Meanwhile, Cantonese is another Chinese dialect (about 70 million native speakers), widely used in Guangdong Province and also the official language of Hong Kong and Macau. Hence, it is worthwhile developing Mandarin norms for the LinguaPix pictures in addition to the Cantonese version. More importantly, the current study adopted a novel approach to the norming procedure, yielding unique and valuable resources for future research.

Most norming studies on picture naming have mainly focused on the dominant name of each picture (i.e., the most frequently produced name, or called the modal name; but see an exception in Wolna et al., 2023). Existing databases mostly provide psycholinguistic characteristics of the dominant names (e.g., Liu et al., 2011; Zhong et al., 2024) or weighted values based on all the modal and non-modal names (e.g., van Hoef et al., 2024). The current study adopted an approach between these two extremes. Besides image agreement, AoA, and concept familiarity of the dominant names, we also collected rating scores on these dimensions for the secondary names (i.e., the second most frequently produced name for a given picture). Hence, our dataset provided richer information on both the dominant and secondary names of pictures than other less frequently produced names. Such a database would greatly facilitate the selection of picture stimuli when researchers need to manipulate or control properties of the dominant and secondary names (e.g., see studies on phonological co-activation and lexical selection in Mädebach et al., 2022 and Peterson & Savoy, 1998). Although the group-level measures of name agreement might not be entirely true for an individual speaker, Balatsou et al. (2022) found that group-level norms did predict individual speakers’ likelihood of using particular picture names when tested more than once, supporting the psychological reality of picture name agreement within individual speakers. Like most megastudies on picture naming, the current study required participants to name each picture only once and reported group-level norms.

With various rating scores on both the dominant and secondary names of pictures, we analyzed the relations between naming performance and rating scores for both names. Moreover, we explored the relations between naming performance for the dominant names and the psycholinguistic properties of their corresponding secondary names and vice versa, as co-activation of alternative names during lexical selection is a widely accepted assumption in models of language production (e.g., Dell, 1986; Levelt et al., 1999). According to competitive accounts of lexical selection, the co-activation of non-target alternatives slows target selection (e.g., Howard et al., 2006; Levelt et al., 1999). Such an assumption predicted that the dominant and secondary names would compete with each other so that stronger activation of the dominant name would slow the production of the secondary name and vice versa. Hence, we hypothesized that the psycholinguistic properties of the dominant names would predict naming performance for both the dominant names and the secondary names, but in different directions. For example, certain properties of the dominant names might be associated with shorter naming latencies for the dominant names but longer latencies for the secondary names. Similarly, we hypothesized that psycholinguistic properties of the secondary names would predict naming performance for the dominant and secondary names in different directions. Separate multiple regression analyses were conducted to identify unique predictors of naming latency and name agreement for the dominant and secondary names, respectively.

## Method

### Participants

Forty-one native Mandarin-speaking adults (mean age = 21.6 years, *SD* = 2.3 years; 6 male) completed the picture naming task with monetary rewards (70 RMB per participant). They were born and had lived in Beijing or Hebei Province (the province surrounding Beijing), and 20 of them received higher education outside Beijing or Hebei Province. Most of them reported speaking English as a second language (L2), with five of them speaking a third language (L3; e.g., French, Korean); only one spoke Japanese as L2 and two reported not speaking an L2.

Another group of 83 native Mandarin-speaking adults (mean age = 21.0 years, *SD* = 1.7 years; 19 male) completed the rating tasks on picture names with monetary rewards (150 RMB per participant). They were born and had lived in Beijing or Hebei Province, and 28 of them received higher education outside Beijing or Hebei Province. All of them reported speaking English as L2, with 12 of them speaking an L3 and three of them speaking a fourth language (L4). Among these 83 participants, 40 completed an additional set of rating tasks on picture content (i.e., familiarity, visual complexity, valence, and arousal; 200 RMB per participant). All participants were neurologically healthy and had normal or corrected-to-normal vision.

### Stimuli

We selected 720 out of the 1547 pictures from the LinguaPix database (Krautz & Keuleers, 2022), based on the picture-naming norms in Cantonese Chinese (https://linguapix.uni-mannheim.de/frontend/web/index.php; Krautz, 2023). As the Cantonese-speaking participants in their study (i.e., southern Chinese recruited from a university in Hong Kong) shared a similar cultural background with our Mandarin-speaking participants (i.e., northern Chinese), we assumed that their ability to recognize the picture content should be comparable. Hence, pictures with a naming accuracy rate below 50% among Cantonese speakers were excluded to reduce the difficulty level of the picture naming task for our participants. When the same Cantonese name was shared by multiple pictures, we compared the content of these pictures and chose to exclude some of them (e.g., 19 pictures sharing the dominant name 花 “flower” with 17 of them being excluded). Pictures of pure colors and shapes were also excluded.

#### The picture naming task

The 720 pictures were divided into two lists, each with 18 blocks (20 pictures per block). Participants of the picture naming task received the two lists in two separate sessions a few days apart, and the order of lists was counterbalanced across participants. They completed the experimental sessions in a quiet room. The stimuli were presented with the E-Prime 3.0 software (https://pstnet.com/products/e-prime/). The order of blocks and the order of pictures within each block were randomized. Five practice trials were provided before the experiment started.

Each trial consisted of a 500-ms fixation, a 3000-ms presentation of one individual picture, and a 1000-ms blank. The participants were required to name the picture as soon as possible (without using articles, adjectives, or full sentences) or to remain silent if they could not recognize the depicted target. They were advised to refrain from hesitating, coughing, sneezing, or yawning. All responses in the 3000-ms intervals were audio-recorded for later processing. The detailed instructions in Chinese are provided in Appendix.

After the picture naming experiment was completed, all the naming responses were transcribed by a research assistant and checked by two student helpers. The dominant and secondary names for each picture were obtained (see details in the Results section below). The research assistant also checked the recordings of all correct responses manually with CheckVocal software (Protopapas, 2007) to extract the naming latency (i.e., reaction time or RT) for each trial. The CheckVocal software automatically detected the speech onset time in each recording, and the research assistant listened to the two halves of the recording (i.e., before and after the speech onset) and, if needed, adjusted the onset time. RT information was not provided for incorrect responses or correct responses with hesitation, self-correction, or other unexpected sounds.

#### The rating tasks

Two sets of rating tasks were adopted (see the instructions in Appendix), focusing on the picture content and the picture names, respectively. The rating tasks on picture content captured four dimensions of the pictures in the LinguaPix database (Krautz & Keuleers, 2022): (1) *familiarity*—how usual or unusual the object presented in the image is to the participant (1 = very unfamiliar, 5 = very familiar), (2) *visual complexity*—the level of detail or complexity that the image depicts (1 = very simple, 5 = very complex), (3) *valence*—the extent to which the image triggers a positive or negative emotion in the participant (1 = very negative emotion, 5 = very positive emotion), and (4) *arousal*—the intensity or strength of an emotional state associated with the image (1 = very not intense, 5 = very intense). These four rating tasks were completed sequentially in separate sessions by each of the 40 participants. The order of pictures was randomized in each task.

Additionally, we included another three rating tasks on the picture names, which were performed by a total of 83 participants. These three tasks were adapted from the study of Liu et al. (2011), which showed significant contributions of image agreement, AoA, and concept familiarity to picture naming latencies. In the first session, participants were asked to rate how closely the picture resembled their mental image given a concept’s name (1 = very remotely, 5 = very closely), i.e., *image agreement*. Two lists of the rating materials were generated by pairing each picture with its dominant name in one list and with its secondary name in the other so that each list contained an equal number of dominant and secondary names. A filler name was used for pictures without a secondary name (e.g., the picture *tiger* was paired with a filler name *熊猫 panda*). Each participant received only one of the two lists and rated each of the pictures once (paired with either its dominant or secondary name). In the second session, participants were asked to estimate their *age of acquisition* (AoA) for each concept name presented alone without pictures (1 = 0–2 years, 2 = 3–4 years, 3 = 5–6 years, 4 = 7–8 years, 5 = 9–10 years, 6 = 11–12 years, 7 = 13 years or above, 8 = unfamiliar name). Among the dominant and secondary names of all the pictures, we identified a total of 1078 unique names, which were divided into two lists. Each participant received only one of the two lists. In the last session, the same lists were used as in the AoA task and participants were required to rate their familiarity with a given concept’s name presented alone without pictures (1 = very unfamiliar, 5 = very familiar), i.e., *concept familiarity*. Half of the participants completed List 1 of the three tasks while the other half completed List 2. The order of picture names was randomized in each list.

All the rating tasks were implemented through the Qualtrics online survey platform (https://www.qualtrics.com/). To ensure data quality, participants were required to meet the research assistant online and complete each rating session within a 1- to 1.5-h interval. Two practice items were provided at the beginning of each list.

## Results

Among the 41 participants of the picture naming task, one dropped out after completing the first session (List 1); another participant was not concentrating on the task in the second session (constantly looking away from the screen), and only data from the first session (List 2) were included in the analysis. This resulted in 40 naming trials to be analyzed for each picture. We further excluded 20 pictures because over 30 participants failed to recognize them correctly (a picture of drumsticks incorrectly recognized as chopsticks, a picture of a compass incorrectly recognized as watch or water meter, no response to pictures of cheese grater and olives, etc.). Below, we report the linguistic norms for the remaining 700 pictures, which spanned 40 semantic categories (see Appendix).

### Name agreement and naming latency

Recordings of all the naming responses were transcribed and categorized into the following: (1) correct responses with valid RT information (85.04%), (2) correct responses without valid RT information (0.78%) due to hesitation, self-correction, or other unexpected sounds, and (3) incorrect or no responses (14.18%). Names that referred to the whole or part of the depicted target were considered correct, and concepts at the superordinate level were also accepted. While the first two types of responses were included when counting correct picture names, only the first type of responses were included in the RT analysis below (see the calculation of naming latencies at the end of this section). The accuracy rate for each picture varied from 27.5% to 100.0% (mean = 85.8%, *SD* = 16.8%). Occasionally participants’ responses included English letters or vocabulary. Those commonly used English loanwords (e.g., CD, T恤 “T-shirt,” U盘 “USB stick”) were included when counting correct picture names, but pure English words (e.g., pen, bike; 11 occurrences only) were not counted although they were considered as correct responses.

We counted the number of different correct names for each picture as well as the frequency of each name. The number of correct names (*no_names*) varied from 1 to 17. The picture with the largest number of names was an anatomical body diagram showing the human muscular system, which was named 人体 “human body,” 肌肉 “muscles,” 人 “human,” 人体结构 “human body structure,” 人体肌肉 “human muscles,” 人体构造 “human body structure,” 人体骨架 “human skeleton,” 人体解剖图 “human anatomy diagram,” 人体模型 “human body model,” 人体图 “human body diagram,” 人体组织 “human tissue,” 人体组织图 “human tissue diagram,” 人体结构图 “human body structure diagram,” 身体 “body,” 骨骼肌 “skeletal muscles,” 躯体 “body,” and 模型 “model.” Different types of relations existed among these names. For example, 人体结构 is equivalent to 人体构造; 骨骼肌 is subordinate to 肌肉; 肌肉 is a part of 身体; 人体模型 and 人体图 share many semantic features but have subtle differences.

For each picture, the name with the highest frequency was identified as the dominant name of that picture; the name with the second highest frequency was taken as the secondary name of that picture. Among the 700 pictures, 13 had two names with the same highest frequency, in which case one of the two names was randomly selected as the dominant name and the other as the secondary name. In addition, 73 of the pictures had two or more names sharing the same second highest frequency, and only one was randomly selected as the secondary name for each picture. There were also 83 pictures with a single name (i.e., the dominant name) and no secondary name. The number of syllables contained in the name was taken as the name length (*first_name_length* and *second_name_length*). Both dominant and secondary names contained one to five syllables, which corresponded to one to five Chinese characters.

**Fig. 1:**
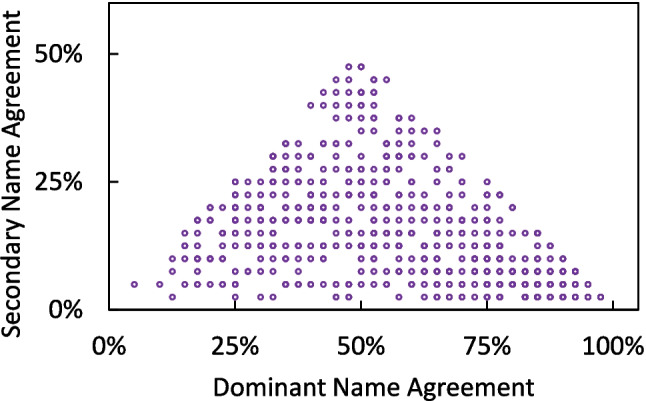
A scatterplot showing the relation between dominant and secondary name agreement

We divided the frequency of dominant name by the total number of naming trials (i.e., 40) to obtain the dominant name agreement (*first_name_agreement*), which ranged from 5.0% to 100.0%. Similarly, the secondary name agreement (*second_name_agreement*) was also calculated, and it ranged from 2.5% to 47.5% (excluding pictures with no secondary name). The descriptive statistics of dominant and secondary name agreement can be found in Table 1. Additionally, Fig. 1 demonstrates the relation between dominant and secondary name agreement. The upper limit of secondary name agreement increases when the dominant name agreement changes from the minimum to 50%, since secondary name agreement cannot exceed dominant name agreement. An inverse relation occurs when the dominant name agreement varies from 50 to 100%, as the sum of dominant and secondary name agreement cannot exceed 1.

**Table 1:** Psycholinguistic norms of the 700 pictures

	No	Minimum	Maximum	Mean	*SD*	Skewness	Kurtosis
accuracy	700	27.5%	100.0%	85.8%	16.8%	− 1.58	1.98
no_names	700	1	17	4.2	2.6	1.16	1.75
*H*_statistic (overall name agreement)	700	0.00	3.64	1.10	0.81	0.48	− 0.46
first_name_agreement	700	5.0%	100.0%	62.9%	24.3%	− 0.15	− 1.07
second_name_agreement	617	2.5%	47.5%	14.7%	10.6%	0.93	0.24
first_name_length	700	1	5	2.2	0.6	0.56	1.66
second_name_length	617	1	5	2.2	0.8	0.57	0.81
first_RT_average	700	784	2313	1325.4	256.6	0.38	− 0.10
second_RT_average	607	796	2685	1491.2	330.8	0.45	0.04
familiarity (max: 5)	700	1.56	5.00	3.63	0.81	− 0.43	− 0.76
visual_complexity (max: 5)	700	1.19	4.82	2.55	0.70	0.60	− 0.21
valence (max: 5)	700	1.53	4.67	3.21	0.39	− 0.40	2.18
arousal (max: 5)	700	1.87	4.11	2.58	0.42	0.82	0.38
first_image_agreement_average (max: 5)	700	3.13	5.00	4.57	0.34	− 1.47	2.13
second_image_agreement_average (max: 5)	617	1.93	5.00	4.36	0.50	− 1.54	2.60
first_AoA_average (max: 8)	700	1.51	6.76	3.58	0.99	0.52	− 0.04
second_AoA_average (max: 8)	615	1.51	6.55	3.63	1.09	0.29	− 0.61
first_concept_familiarity_average (max: 5)	700	2.90	5.00	4.52	0.37	− 1.27	1.63
second_concept_familiarity_average (max: 5)	615	2.78	5.00	4.47	0.41	− 1.27	1.41

In addition to the dominant and secondary name agreement, we calculated the *H*-statistic based on the number of different correct names for a given picture as well as the proportion of each name among all correct responses to the picture (see the formula in Snodgrass & Vanderwart, 1980). Unlike the percentage score for the dominant and secondary name agreement, *H*-statistic integrates the probability of not only the dominant and secondary names but also the other alternative names, so it is commonly used as an overall measurement of name agreement. When a picture has a single correct name, the *H*-statistic is 0. A larger *H*-statistic indicates lower name agreement. Note that only correct responses are included in the calculation of *H*-statistic regardless of the accuracy rate (e.g., a single-name picture with 100% accuracy rate shares the same *H*-statistic as another single-name picture with 90% accuracy rate).

We categorized the semantic relation between dominant and secondary names into the following: (1) equivalent—the secondary name is equivalent to the dominant name (e.g., 斧头 “axe” and 斧子 “axe”), 25.86%; (2) superordinate—the secondary name is a more general category than the dominant name (e.g., 救护车 “ambulance” and 车 “vehicle”), 22.29%; (3) subordinate—the secondary name is a more specific category than the dominant name (e.g., 钱 “money” and 纸币 “banknotes”), 20.14%; (4) multi-focus—the secondary name reflects a focus of the speaker other than the dominant name (e.g., 烟灰缸 “ashtray” and 烟头 “cigarette butts”), 9.00%; (5) overlap—the secondary name refers to a similar but different concept than the dominant name (e.g., 闹钟 “alarm clock” and 计时器 “timer”), 10.86%. The rest of the pictures (11.85%) had no secondary name and thus were not included in these categories. The initial categorization was performed by the research assistant and one student helper independently, and inconsistencies were resolved through discussion between the research assistant and the corresponding author (for 17.1% of the pictures). This categorical information is listed under the variable *name_relation* in the current database.

We further obtained valid RT information from 85.04% of the trials and excluded extreme values that exceeded 2.5 *SD* of each item mean (1.58%). The average naming latency was calculated for the dominant and secondary names of each picture (*first_RT_average* and *second_RT_average*), respectively. Besides the 83 pictures with no secondary name, a further 10 pictures had no valid RT data for the secondary name. RT information for the other alternative names is available in the trial-level data file in the online repository.

### Rating results and validity checks

The three rating tasks on picture names (i.e., image agreement, AoA, and concept familiarity of the dominant and secondary names) were completed by 83 Mandarin speakers, with 41 completing List 1 of the three tasks and 42 completing List 2.^1^ We excluded extreme values that exceeded 2.5 *SD* of each item mean and counted the number of remaining data points for each participant and item. One participant of List 1 and two participants of List 2 were identified as outliers, as their total number of remaining data points was lower than 2.5 *SD* of the mean number of remaining data points. Hence, all of the data for these three participants were excluded, and 40 participants remained for each list. The average number of remaining data points in the three tasks was 39.1, 39.6, and 39.3 for the dominant name and 39.2, 39.7, and 39.5 for the secondary name. The average rating scores were calculated for each item and listed under *first_image_agreement_average*, *first_AoA_average*, *first_concept_familiarity_average*, *second_image_agreement_average*, *second_AoA_average*, and *second_concept_familiarity_average*, respectively. Examples of picture names with low AoA included 手 “hand” and 狗 “dog”; those with high AoA included 学士帽 “graduation hat” and 睫毛膏 “mascara.” Examples of picture names with low concept familiarity included 修女 “nun” and 火烈鸟 “flamingo”; those with high concept familiarity included 手机 “mobile phone” and 钥匙 “key.”

The same data trimming procedure was applied to the rating scores on picture content (i.e., *familiarity*, *visual_complexity*, *valence*, and *arousal*), and 38 out of the 40 participants remained, with two outliers being excluded. The average number of remaining data points in these four tasks was 37.6, 37.7, 37.8, and 37.9. Examples of pictures with negative valence included 小偷 “thief” and 蛇 “snake”; those with positive valence included 猫 “cat” and 向日葵 “sunflower.” Examples of pictures with low arousal included 线 “thread” and 石头 “stone”; those with high arousal included 小丑 “clown” and 花园 “garden.”

To check the rating consistency across the sample of Mandarin speakers, we randomly divided the sample into two halves (i.e., around 20 participants each) for each rating task, and the split-half correlations of the rating scores were all above 0.8 (familiarity 0.958, visual complexity 0.918, valence 0.910, arousal 0.811, image agreement 0.970, AoA 0.950, and concept familiarity 0.914). For the four dimensions of picture content, we also extracted the rating scores of Cantonese speakers from the LinguaPix database (https://linguapix.uni-mannheim.de/frontend/web/index.php; Krautz, 2023) and analyzed the rating results from the two groups of Chinese (i.e., northern and southern Chinese). The between-group correlations of the rating scores were above 0.7 for visual complexity (0.717), valence (0.792), and arousal (0.703), while the correlation was moderate for familiarity (0.416). These results suggest that visual and affective attributes of pictures are relatively stable across people from similar cultural backgrounds but familiarity is reliant on exposure and probably more sensitive to regional differences.

We further checked the validity of picture name ratings against previous studies. Liu et al. (2011) provided naming norms for 435 black-and-white line drawings in Mandarin Chinese, including adult ratings on the AoA of dominant names. For the 285 picture names that were included in our current database, the between-study correlation of AoA ratings was 0.794. Xu et al. (2021) also provided AoA norms for 19,716 Chinese words by Mandarin speakers, which included 492 of the picture names in our database. The correlation of AoA ratings between Xu et al. (2021) and the current study was 0.797. As for concept familiarity of the picture names, our rating results correlated strongly with subjective estimation of word frequency in Liu et al. (2011), *r* = 0.731, but weakly with log-transformed word frequency based on film subtitles (Cai & Brysbaert, 2010), *r* = 0.213 (*N* = 838). One possible explanation is that the corpus of film subtitles in Chinese (Cai & Brysbaert, 2010) was based on films produced worldwide, including translated subtitles, and thus was unable to capture regional differences in people’s exposure to the objects depicted in LinguaPix pictures. Subjective ratings on word frequency or familiarity could be quite different from objective lexical frequency of this type in the case of common object names. Hence, we added objective word frequency to our database as supplementary information, but we used the rated concept familiarity of the picture names in the regression analyses below.

### Relations between name agreement and other variables

**Fig. 2:**
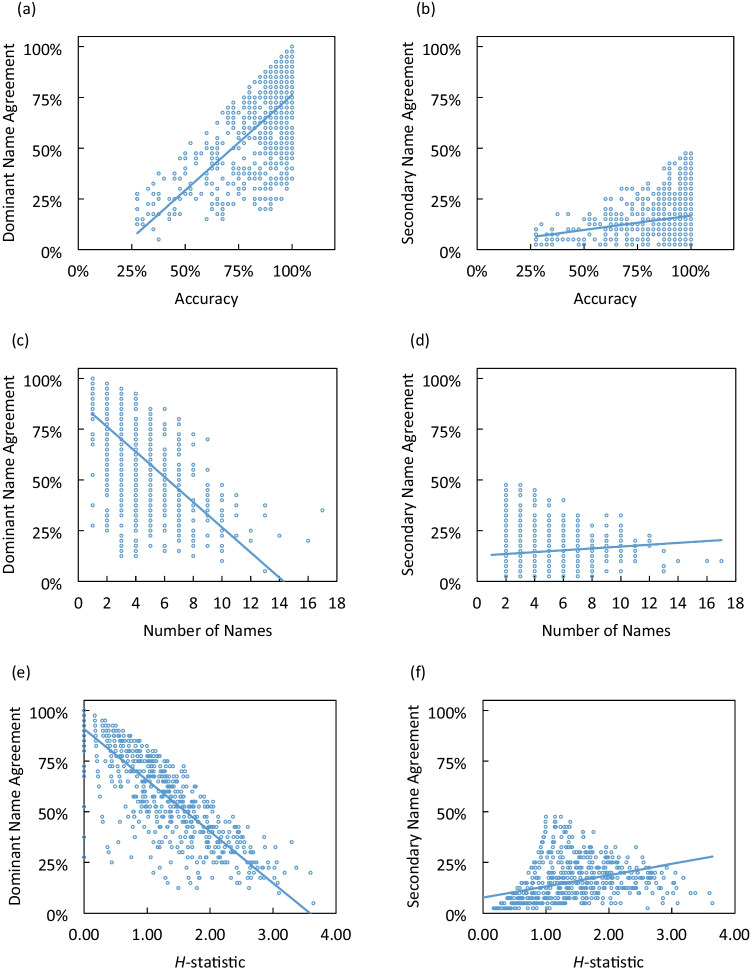
Scatterplots showing the relations between name agreement and other variables: accuracy (**a**, **b**), number of names (**c**, **d**), and *H*-statistic (**e**, **f**)

Table 2 shows the zero-order correlations between name agreement and other variables. Accuracy was positively correlated with both dominant and secondary name agreement (Fig. 2a, b). As the number of names increased, dominant name agreement declined dramatically (Fig. 2c). In contrast, there was a weak positive correlation between the number of names and secondary name agreement (Fig. 2d). Similarly, a larger *H*-statistic was associated with lower dominant name agreement but higher secondary name agreement (Fig. 2e, f). Among the four dimensions of ratings on the pictures, familiarity and arousal were positively correlated with dominant name agreement while visual complexity and valence were not. Only familiarity was significantly correlated with secondary name agreement. Additionally, all three dimensions of ratings on the dominant name were significantly correlated with dominant name agreement, and one of them (i.e., *first_concept_familiarity_average*) was also weakly correlated with secondary name agreement. The three dimensions of ratings on the secondary name were significantly correlated with secondary name agreement, but not with dominant name agreement.

**Table 2:** Zero-order correlations between name agreement, naming latency, and other variables

	first_name_agreement	second_name_agreement	first_RT_average	second_RT_average
accuracy	0.649^**^	0.226^**^	− 0.693^**^	− 0.557^**^
no_names	− 0.659^**^	0.106^**^	0.472^**^	0.194^**^
*H*_statistic (overall name agreement)	− 0.842^**^	0.388^**^	0.536^**^	0.179^**^
familiarity	0.140^**^	0.141^**^	− 0.274^**^	− 0.294^**^
visual_complexity	0.045	− 0.047	0.049	0.108^**^
valence	0.015	0.068	− 0.096^*^	− 0.017
arousal	0.182^**^	0.044	− 0.201^**^	− 0.098^*^
first_image_agreement_average	0.477^**^	0.031	− 0.540^**^	− 0.362^**^
first_AoA_average	− 0.208^**^	− 0.065	0.239^**^	0.264^**^
first_concept_familiarity_average	0.135^**^	0.089^*^	− 0.160^**^	− 0.221^**^
second_image_agreement_average	0.067	0.230^**^	− 0.275^**^	− 0.309^**^
second_AoA_average	− 0.069	− 0.169^**^	0.147^**^	0.153^**^
second_concept_familiarity_average	0.003	0.221^**^	− 0.109^**^	− 0.121^**^

^*^p < 0.05, ^**^p < 0.01

In order to identify unique predictors of name agreement while controlling for other variables, we conducted multiple regression analyses in which different groups of variables were entered by block. For *first_name_agreement* (*N* = 615), the following variables were entered: (1) Block 1 included general control variables: *accuracy*, *no_names*, *first_name_length_two,*^2^*first_name_length_above_two*; (2) Block 2 included the four dimensions of ratings on the pictures: *familiarity*, *visual_complexity*, *valence*, and *arousal*; (3) Block 3 included the three dimensions of ratings on the dominant name: *first_image_agreement_average*, *first_AoA_average*, and *first_concept_familiarity_average*; and (4) Block 4 included the three dimensions of ratings on the secondary name: *second_image_agreement_average*, *second_AoA_average*, and *second_concept_familiarity_average*. While all the control variables were directly entered in Block 1, the other blocks adopted the forward selection method so as to identify the dimensions of ratings that uniquely predicted dominant name agreement beyond control variables.

Table 3 shows the results of the multiple regression analysis on dominant name agreement. Higher dominant name agreement was associated with higher accuracy, smaller number of names, and higher image agreement and concept familiarity of the dominant name. In addition, higher dominant name agreement was associated with lower image agreement and concept familiarity of the secondary name. Note that the zero-order correlations between dominant name agreement and these two ratings of the secondary name were not significant. Their relations were revealed after controlling for other variables. On the other hand, familiarity,^3^ arousal, and AoA of the dominant name had no unique contribution to dominant name agreement, although their zero-order correlations with dominant name agreement were significant. Overall, the unique predictors in the model accounted for 72.0% of the variance in dominant name agreement (*R*^2^ = 0.720, adjusted* R*^2^ = 0.716; *F*(9, 605) = 172.85, *p* < 0.001).

**Table 3:** Parameter estimates, statistical significance, and degree of multicollinearity in the multiple regression analysis on *dominant name agreement*

	Standardized *β*	*t*	*p*	Tolerance	VIF
accuracy	0.567	20.910	< 0.001	0.630	1.588
no_names	− 0.502	− 22.344	< 0.001	0.918	1.089
first_name_length_two	0.018	0.508	0.612	0.367	2.724
first_name_length_above_two	0.035	0.958	0.338	0.350	2.859
familiarity_average	− 0.048	− 1.356	0.176	0.372	2.685
first_image_agreement_average	0.125	4.258	< 0.001	0.535	1.870
first_concept_familiarity_average	0.111	3.328	< 0.001	0.419	2.384
second_image_agreement_average	− 0.141	− 5.944	< 0.001	0.824	1.214
second_concept_familiarity_average	− 0.126	− 4.691	< 0.001	0.639	1.565

A similar multiple regression analysis was conducted on *second_name_agreement* (*N* = 615), with the three dimensions of ratings on the secondary name entered in Block 3 while those on the dominant name were entered in Block 4. As shown in Table 4, higher secondary name agreement was associated with higher accuracy, larger number of names, and higher image agreement and concept familiarity of that name. Secondary names containing two syllables tended to have higher name agreement than monosyllabic names, while no significant difference was found between names containing one or more than two syllables. Interestingly, the zero-order correlation between concept familiarity of the dominant name and secondary name agreement was positive, but their relation was reversed to negative after controlling for other variables. Image agreement of the dominant name also negatively predicted secondary name agreement in the model. On the other hand, AoA of the secondary name had no unique contribution to secondary name agreement, although their zero-order correlation was significant. Overall, the unique predictors in the model accounted for 17.7% of the variance in secondary name agreement (*R*^2^ = 0.177, adjusted* R*^2^ = 0.166; *F*(8, 606) = 16.27, *p* < 0.001).

**Table 4:** Parameter estimates, statistical significance, and degree of multicollinearity in the multiple regression analysis on *secondary name agreement*

	Standardized *β*	*t*	*p*	Tolerance	VIF
accuracy	0.252	5.465	< 0.001	0.637	1.570
no_names	0.093	2.435	0.015	0.930	1.076
second_name_length_two	0.148	2.736	0.006	0.463	2.158
second_name_length_above_two	0.062	1.087	0.278	0.420	2.380
second_image_agreement_average	0.272	6.720	< 0.001	0.830	1.204
second_concept_familiarity_average	0.266	5.843	< 0.001	0.653	1.531
first_concept_familiarity_average	− 0.170	− 3.667	< 0.001	0.634	1.578
first_image_agreement_average	− 0.167	− 3.613	< 0.001	0.634	1.578

### Relations between naming latency and other variables

As shown in Table 2, the zero-order correlations between dominant name RT and other variables were all significant except for visual complexity. Larger number of names, larger *H*-statistic, and higher AoA of the dominant and secondary name tended to slow dominant name production (i.e., longer RT), while other variables tended to facilitate it (i.e., shorter RT). As for secondary name RT, a highly similar pattern was found except that secondary name RT was not associated with valence but tended to be longer with higher visual complexity of the picture.

We also conducted multiple regression analyses on *first_RT_average* (*N* = 615) and *second_RT_average* (*N* = 605). The same sets of variables were entered by block as in the regression analyses on name agreement except that number of names was replaced by the *H*-statistic so as to have better control over the influence of name agreement on naming latency. The results are shown in Tables 5 and 6, respectively. Factors that significantly and uniquely predicted dominant name RT included accuracy, *H*-statistic, dominant name length, and visual complexity, as well as image agreement of the dominant and secondary names. Although the zero-order correlation between dominant name RT and visual complexity was not significant, dominant name RT tended to be longer with higher visual complexity of the picture after controlling for other variables. Dominant names containing two syllables tended to have shorter naming latencies than monosyllabic names, while no significant difference was found between names containing one or more than two syllables. More importantly, the relation between image agreement of the secondary name and dominant name RT remained negative (i.e., facilitation on dominant name production) after controlling for other variables. Familiarity, valence,^4^ and arousal as well as AoA and concept familiarity of the dominant and secondary names had no unique contribution to dominant name RT, although their zero-order correlations with dominant name RT were significant. Overall, the unique predictors in the model accounted for 63.4% of the variance in dominant name RT (*R*^2^ = 0.634, adjusted* R*^2^ = 0.629; *F*(9, 605) = 116.57, *p* < 0.001).

**Table 5:** Parameter estimates, statistical significance, and degree of multicollinearity in the multiple regression analysis on *dominant name RT*

	Standardized *β*	*t*	*p*	Tolerance	VIF
accuracy	− 0.532	− 18.170	< 0.001	0.704	1.420
*H*_statistic	0.331	12.507	< 0.001	0.862	1.160
first_name_length_two	− 0.094	− 2.341	0.020	0.377	2.653
first_name_length_above_two	− 0.072	− 1.751	0.080	0.363	2.758
visual_complexity_average	0.101	3.411	< 0.001	0.689	1.452
arousal_average	− 0.059	− 1.954	0.051	0.657	1.522
valence_average	− 0.044	− 1.767	0.078	0.972	1.029
first_image_agreement_average	− 0.097	− 3.125	0.002	0.626	1.599
second_image_agreement_average	− 0.106	− 3.972	< 0.001	0.855	1.170

**Table 6:** Parameter estimates, statistical significance, and degree of multicollinearity in the multiple regression analysis on *secondary name RT*

	Standardized *β*	*t*	*p*	Tolerance	VIF
accuracy	− 0.453	− 12.004	< 0.001	0.757	1.321
*H*_statistic	0.080	2.348	0.019	0.927	1.079
second_name_length_two	− 0.136	− 2.668	0.008	0.415	2.407
second_name_length_above_two	− 0.143	− 2.564	0.011	0.346	2.889
familiarity_average	− 0.047	− 1.266	0.206	0.771	1.298
second_image_agreement_average	− 0.182	− 5.115	< 0.001	0.854	1.171
second_AoA_average	0.120	2.938	0.003	0.642	1.558

As shown in Table 6, factors that significantly and uniquely predicted secondary name RT included accuracy, *H*-statistic, secondary name length, and image agreement and AoA of the secondary name. Secondary names containing two or more than two syllables tended to have shorter naming latencies than monosyllabic names. Familiarity,^5^ visual complexity, arousal, all ratings on the dominant name, and concept familiarity of the secondary name had no unique contribution to secondary name RT although their zero-order correlations with secondary name RT were significant. Overall, the unique predictors in the model accounted for 35.7% of the variance in secondary name RT (*R*^2^ = 0.357, adjusted* R*^2^ = 0.350; *F*(7, 597) = 47.43, *p* < 0.001).

## Discussion

The current study provided psycholinguistic norms for 700 color photographs from the LinguaPix database in Mandarin Chinese, including name agreement, naming latency, name length, image agreement, AoA, and concept familiarity of the dominant and secondary names, as well as overall accuracy, number of names, *H*-statistic, familiarity, visual complexity, valence, and arousal of the pictures. Existing databases have provided Mandarin norms for both black-and-white and colored line drawings (Bates et al., 2003; Duñabeitia et al., 2022; Liu et al., 2011), and the current study constituted a notable addition to this line of research by creating Mandarin norms for carefully edited color photographs. More importantly, we provided various rating scores not only on the dominant names of the pictures but also on their secondary names, which were not available in previous Mandarin databases. Hence, the current database greatly increases the diversity of stimuli as well as the type and amount of name-related information for picture naming studies in Chinese. Below, we present the most relevant findings observed in this study.

### Comparisons between LinguaPix (German) and the current results

In the LinguaPix study of German speakers (Krautz & Keuleers, 2022), participants’ ratings of the pictures (i.e., familiarity, visual complexity, valence, and arousal) were all positively correlated with name agreement, suggesting that pictures with higher name agreement tended to be more familiar to participants, visually more complex, and associated with more positive and more intense emotions. In the current study, positive correlations were also observed between dominant name agreement and two picture ratings (i.e., familiarity and arousal), but Mandarin speakers showed no significant correlations between dominant name agreement and the other two picture ratings (i.e., visual complexity and valence). One possible explanation is that the correlations between name agreement and two dimensions of the rating scores were significant but rather weak among German speakers (visual complexity: *r* = 0.077; valence: *r* = 0.113), which might have failed to be captured in the current study with a smaller set of pictures (i.e., 700 out of 1547). As for secondary name agreement in the current study, it was associated with only one dimension of the picture rating scores (i.e., familiarity).

In terms of naming latency, German speakers took longer to respond to pictures that were less familiar to them, visually more complex, and associated with more negative and less intense emotions (Krautz & Keuleers, 2022). The same pattern was observed for dominant and secondary name RT among Mandarin speakers, although the zero-order correlation between dominant name RT and visual complexity and that between secondary name RT and valence were not significant.

In addition to the rating scores on picture content, the current study also provided three dimensions of ratings (i.e., image agreement, AoA, and concept familiarity) on both the dominant and secondary names, which were not included in the existing LinguaPix database (Krautz, 2023). In order to identify unique predictors of name agreement and naming latency, the various rating scores on picture content and picture names were entered into multiple regression analyses by block, with the forward selection method, while controlling for other variables. At least one dimension of ratings on picture content remained in the models of dominant name agreement, dominant name RT, and secondary name RT after controlling for accuracy and other factors. However, most of these picture ratings became nonsignificant predictors after the ratings on picture names were entered in the subsequent blocks. These results indicated that name agreement and naming latency, when calculated separately for a particular picture name like in the current study, might be better predicted by attributes of that name rather than those of the picture content (see also the elusive effects of visual complexity, valence, and arousal ratings in Alario et al., 2004; Blackett et al., 2017; Liu et al., 2011; White et al., 2016; Zhong et al., 2024).

### The dominant and secondary names

We entered participants’ ratings of the dominant and secondary names in the last two blocks of the multiple regression analyses of name agreement and naming latency, in order to examine whether dominant name agreement and RT would be affected by secondary name ratings or vice versa. A relation of mutual competition appeared in the multiple regression models of name agreement. Dominant name agreement was positively associated with image agreement and concept familiarity of the dominant name and negatively with image agreement and concept familiarity of the secondary name. On the other hand, secondary name agreement was positively associated with image agreement and concept familiarity of the secondary name and negatively with image agreement and concept familiarity of the dominant name. Such mutual competition between the dominant and secondary names in terms of name agreement was due to the fact that participants could produce only one name for each picture. The more likely they would produce the dominant name, the less likely the secondary name; and vice versa. Note that the negative relations between name agreement and rating scores were revealed in the multiple regression models after controlling for other variables but not reflected by their zero-order correlations. It is thus important to conduct both correlation and regression analyses when investigating potential factors influencing picture naming performance.

As for naming latency, no mutual competition was observed between the dominant and secondary names, which was inconsistent with the predictions of competitive accounts of lexical selection (e.g., Howard et al., 2006; Levelt et al., 1999; see also noncompetitive accounts of lexical selection in Mahon et al., 2007; Oppenheim et al., 2010; Staub et al., 2015). Shorter dominant name RT was associated with higher image agreement of both the dominant and secondary names. The zero-order correlation between dominant name RT and image agreement of the secondary name was significantly negative, and this negative relation remained in the multiple regression model of dominant name RT. This means that higher image agreement of the secondary name uniquely predicted shorter dominant name RT beyond the effects of control variables and other rating scores. However, competitive accounts of lexical selection (e.g., Howard et al., 2006; Levelt et al., 1999) predicted that the secondary name with higher image agreement would become a stronger competitor and thus further delay dominant name production, which was opposite to the current observation. On the other hand, shorter secondary name RT was significantly associated with higher image agreement and lower AoA of the secondary name but not with any ratings of the dominant name.

Recently, Oppenheim (2024) conducted a timed norming study with 100 English speakers and 520 line drawings from the International Picture Naming Project (Bates et al., 2003). The dominant and secondary names of the pictures were identified, and shorter dominant name RT was found to be associated with higher secondary name agreement. Computational simulations demonstrated that when assuming an absolute threshold for target selection (i.e., independent of non-target co-activation), strong alternatives would prevent slow target responses from emerging and reduce the observed dominant name RT. Such a noncompetitive mechanism of lexical selection could also explain the relation between dominant name RT and image agreement of the secondary name in the current study, which remains to be verified by computational simulations in future studies.

Last but not least, a disadvantage was observed for monosyllabic picture names. Dominant names containing two syllables tended to have shorter naming latencies than monosyllabic names. Secondary names containing two syllables tended to have higher name agreement and shorter naming latencies than monosyllabic names, and those containing more than two syllables also tended to have shorter naming latencies. This counterintuitive effect of name length echoed the finding of a French picture naming study that trisyllabic names tended to have shorter naming latencies than monosyllabic or disyllabic names (Alario et al., 2004). However, a similar pattern was not found in several previous studies (e.g., Bates et al., 2003; Dell’Acqua et al., 2000; Liu et al., 2011; Snodgrass & Yuditsky, 1996). The mixed findings could be partly due to the relatively small number of items in picture naming studies and the uneven distribution of name length. A more robust U-shaped relation between word length and lexical decision latency has been reported in the field of visual word recognition (e.g., Ferrand et al., 2010; New et al., 2006; Tsang et al., 2018; Yap & Balota, 2009), with much larger stimulus sets (i.e., tens of thousands of words). Hence, increasing the number of items and controlling well for other major factors may benefit further investigation of the name length effect in picture naming studies.

## Conclusions

The current study is among the first attempts to establish psycholinguistic norms for both the dominant and secondary names of pictures. We provided psycholinguistic norms for 700 color photographs from the LinguaPix database in Mandarin Chinese, including name agreement, naming latency, name length, image agreement, AoA, and concept familiarity of the dominant and secondary names, as well as overall accuracy, number of names, *H*-statistic, familiarity, visual complexity, valence, and arousal of the pictures. Such a database will provide valuable resources and greatly facilitate stimuli selection for psycholinguistic studies on Chinese word production. Researchers can manipulate not only common psycholinguistic properties of the dominant picture name but also those of the secondary name and the relations between the dominant and secondary names (e.g., type of semantic relation, ratio of secondary name agreement to dominant one). The database is freely available in the Open Science Framework repository (https://osf.io/5rphx/).

## Data Availability

The data that support the findings of the current study are available in the Open Science Framework repository (https://osf.io/5rphx/).

## Appendix

### Instructions for the picture naming task with English translations

稍后您将看到一系列图片, 请在图片出现后说出图片的名称。每张图片在3秒钟后会自动消失, 在此期间电脑会自动录入您所说出的名称, 无需按键。 “You will be presented a series of pictures on the screen. Please name the pictures upon seeing them. Each picture will be shown for 3 s and then automatically disappear. The microphone will automatically record your answers during this period. There is no need for you to press any buttons.”

请注意: “Important:”

请勿使用任何冠词 (比如, 一个苹果), 直接命名 (比如, 苹果) “don’t use articles (e.g., an apple) but name the item itself (e.g., apple).”

请勿添加任何形容词 (比如, 绿色的苹果), 直接命名 (比如, 苹果) “don’t use adjectives to describe the items (e.g., green apple) but name the item itself (e.g., apple).”

请勿使用句式表达 (比如, 这是一个苹果), 直接命名 (比如, 苹果) “don’t use full sentences (e.g., It is an apple) but name the item itself (e.g., apple).”

请尽量不要发出思考声音 (比如 “Hmm…”, “Err…” 等) “try to avoid using hesitation devices (e.g., hmm, err), if possible.”

请尽量避免咳嗽声、喷嚏声、哈欠声等等 “try to avoid coughing, sneezing, yawning, if possible.”

请尽量用脑海中的第一印象进行明确的表达 “be specific but use the first word that comes to your mind.”

请尽量快速地反应 “name the items as fast as possible.”

如果无法辨识图片的内容, 请保持沉默。 “if you don’t know the name of the item, don’t say anything.”

**Table 7:** Instructions for the rating tasks with English translations

Tasks	Instructions	Options
familiarity	稍后您将看到一系列图片逐一呈现。请选择每一张图片的内容对于您而言的熟悉常见程度。 “You will be presented a series of pictures one by one. Please rate how usual or unusual an object presented on the picture is in your realm of experience.”	1 = 非常不熟悉常见 “very unfamiliar” 2 = 不熟悉常见 “unfamiliar” 3 = 一般 “neutral” 4 = 熟悉常见 “familiar” 5 = 非常熟悉常见 “very familiar”
visual complexity	稍后您将看到一系列图片逐一呈现。请选择每一张图片的视觉复杂性, 即图片描绘内容的细节及复杂程度。 “You will be presented a series of pictures one by one. Please rate the amount of detail or intricacy a given picture depicts.”	1 = 视觉上非常简单 “visually very simple” 2 = 视觉上较简单 “visually simple” 3 = 一般 “neutral” 4 = 视觉上较复杂 “visually complex” 5 = 视觉上非常复杂 “visually very complex”
valence	稍后您将看到一系列图片逐一呈现。请选择每一张图片唤起您积极或消极情绪的程度。 “You will be presented a series of pictures one by one. Please rate the extent to which a given picture evokes a positive or negative emotion.”	1 = 非常负面消极 “very negative” 2 = 负面消极 “negative” 3 = 一般 “neutral” 4 = 正面积极 “positive” 5 = 非常正面积极 “very positive”
arousal	稍后您将看到一系列图片逐一呈现。请选择每一张图片唤起您情绪的强烈程度。 “You will be presented a series of pictures one by one. Please rate the intensity or strength of an emotional state associated with a given picture.”	1 = 非常不强烈 “very not intense” 2 = 不强烈 “not intense” 3 = 一般 “neutral” 4 = 强烈 “intense” 5 = 非常强烈 “very intense”
image agreement	稍后您将看到一系列概念及其对应图片逐一呈现。请选择您对此概念的印象接近其对应图片的程度, 例如您对 “苹果”的印象是否接近图片中的样子。 “You will be presented a series of concept names and their corresponding pictures one by one. Please rate how closely each picture resembles your mental image of the concept, e.g., whether the picture resembles your mental image of apple.”	1 = 非常不接近 “very low agreement” 2 = 不接近 “low agreement” 3 = 一般 “neutral” 4 = 接近 “high agreement” 5 = 非常接近 “very high agreement”
age of acquisition (AoA)	稍后您将看到一系列概念的名称逐一呈现。请选择您大致是在哪一个年龄阶段习得该名称, 如果不确定可以进行估计。 “You will be presented a series of concept names one by one. Please estimate the age range when you first learned a given concept name.”	1 = 0–2 岁 “0–2 years” 2 = 3–4 岁 “3–4 years” 3 = 5–6 岁 “5–6 years” 4 = 7–8 岁 “7–8 years” 5 = 9–10 岁 “9–10 years” 6 = 11–12 岁 “11–12 years” 7 = 13 岁或以上 “13 years or older” 8 = 不熟悉此名称 “unfamiliar name”
concept familiarity	稍后您将看到一系列概念的名称逐一呈现。请选择您对每一概念的熟悉程度。 “You will be presented a series of concept names one by one. Please rate your familiarity with a given concept name.”	1 = 非常不熟悉 “very unfamiliar” 2 = 不熟悉 “unfamiliar” 3 = 一般 “neutral” 4 = 熟悉 “familiar” 5 = 非常熟悉 “very familiar”

**Table 8:** Distribution of semantic categories in the 700 pictures

No	Semantic category	No. of pictures	No	Semantic category	No. of pictures
1	clothing and accessories	64	21	parts of a house	12
2	food	44	22	bathroom appliances	11
3	kitchen utensils	41	23	insects	10
4	animals	39	24	buildings	9
5	body parts	34	25	marine life	9
6	stationary	30	26	musical instruments	9
7	vegetables	30	27	beverages	8
8	electronic appliances	28	28	household chores	8
9	sports equipment	28	29	medical accessories	8
10	games and toys	27	30	repositories	8
11	household items	26	31	flowers	7
12	vehicles	26	32	weapons	7
13	beauty products and tools	23	33	jewellery	6
14	home furnishings	21	34	materials	6
15	tools	21	35	nuts	6
16	furniture	20	36	celebrations	5
17	professions	19	37	mystical creatures	3
18	fruit	18	38	animals body parts	2
19	bird	12	39	garden tools	2
20	nature	12	40	trees	1

## Footnotes

1 Due to a coding error, two secondary names (i.e., 模型 “model” and 台历 “desk calendar”) were not included in the rating lists of AoA and concept familiarity. Instead, two other secondary names (i.e., 油画 “oil painting” and 啤酒瓶 “beer bottle”) were included in both lists and rated by all the participants.

2 We used two dummy variables (i.e., *first_name_length_two* and *first_name_length_above_two*) to categorize the dominant name length into one syllable, two syllables, or more than two syllables (i.e., 3 ~ 5 syllables). This is because only nine pictures had a dominant name with four or five syllables. A similar way of coding was adopted for the secondary name length below.

3 Familiarity was entered in Block 2 of the multiple regression analysis on dominant name agreement via the forward selection method, but it became nonsignificant after other variables were entered in Blocks 3 and 4.

4 Valence and arousal were entered in Block 2 of the multiple regression analysis on dominant name RT via the forward selection method, but they became nonsignificant after other variables were entered in Blocks 3 and 4.

5 Familiarity was entered in Block 2 of the multiple regression analysis on secondary name RT via the forward selection method, but it became nonsignificant after other variables were entered in Block 3.

## Code availability

The SPSS syntax and output of data analysis are available in the Open Science Framework repository (https://osf.io/5rphx/).
